# A Rare Case of HHV-8 Associated Hemophagocytic Lymphohistiocytosis in a Stable HIV Patient

**DOI:** 10.1155/2019/3297463

**Published:** 2019-04-28

**Authors:** Nonso Osakwe, Diane Johnson, Natalie Klein, Dalia Abdel Azim

**Affiliations:** ^1^Department of Infectious Disease, NYU Winthrop Hospital, Mineola, NY, USA; ^2^Department of Pathology, NYU Winthrop Hospital, Mineola, NY, USA

## Abstract

**Background:**

Hemophagocytic lymphohistiocytosis (HLH) is a rare condition associated with viral infections including HIV. Cases have been reported mainly in advanced HIV/AIDS. This is a rare case that reports HLH associated with human herpes virus-8 (HHV-8) associated multicentric Castleman disease in a stable HIV patient.

**Case Presentation:**

A 70-year-old Asian male patient with history of stable HIV on medications with CD 4 cell count above 200 presented with cough and fever and was initially treated for pneumonia as an outpatient. Persisting symptoms prompted presentation to the hospital. The patient was found to have anemia which persisted despite repeated transfusion of packed red cells. A bone marrow biopsy to investigate anemia revealed hemophagocytosis. A CT scan revealed multiple enlarged lymph nodes and hepatosplenomegaly. An excisional lymph node biopsy revealed HHV-8 associated multicentric Castleman disease. The patient deteriorated despite initiation of treatment.

**Conclusion:**

HLH can occur at any stage of HIV, rapid diagnosis to identify possible underlying reactive infectious etiology and prompt initiation of treatment is crucial to survival.

## 1. Introduction

Hemophagocytic lymphohistiocytosis (HLH) is a rare condition that was first described in 1939 by Scott and Robb-Smith [1]. Its association with viral infection was first described in 1979 [2]. A primary form usually associated with an underlying genetic abnormality and a secondary form associated with underlying infection, malignancies, and immunodeficiency disorders like HIV/AIDS have been described [1, 3]. This condition has been described as a syndrome of excessive inflammation and tissue destruction due to abnormal immune activation and excessive inflammation [4]. HLH has been reported in patients with acute HIV infection and advanced AIDS [5] but we only found few literatures with reports of HLH in stable HIV patients on HAART [1, 2]. There are only a few cases of HLH related to human herpes virus-8 (HHV-8) associated multicentric Castleman disease and Kaposi sarcoma described [6].

## 2. Case

**Figure 1:**
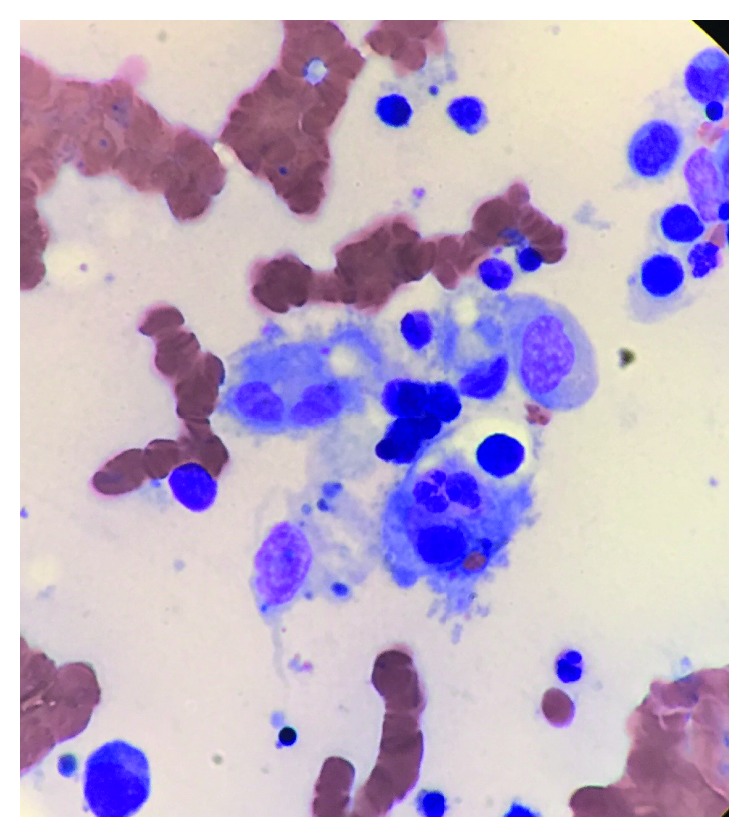
Phagocytosis of hematopoietic cells by macrophages seen in hemophagocytic lymphohistiocytosis (HLH).

**Figure 2:**
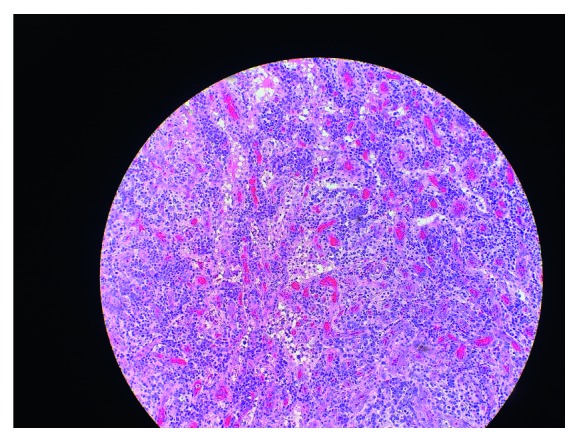
Histological appearance of axillary lymph node showing HHV-8 associated multicentric Castleman disease with plasmablastic aggregates and Kaposi sarcoma on CD 138 stain.

A 70-year-old Asian male presents to the emergency room with a 2-week history of productive cough and fever. Temperature usually as high as 102°F occurred mostly at night time with associated chills. Cough was productive of whitish sputum without blood. He had seen his primary care doctor as an outpatient and was prescribed 5 days of amoxicillin/clavulanic acid for presumed community-acquired pneumonia. His history is significant for HIV diagnosed about 8 months ago with CD4 lymphocytes count of 121 cells/mm^3^ and an HIV viral load of 109,720 copies/mL. At that time, he was started on atovaquone for *Pneumocystis jirovecii* pneumonia prophylaxis. He has been on dolutegravir/emtricitabine/tenofovir alafenamide with his most recent CD4 lymphocyte counts above 200 and HIV viral load 20. Physical examination was positive for splenomegaly but otherwise unremarkable. His blood tests showed hemoglobin of 6.5 g/dL and platelet 81,000 × 106. Chest X-ray showed no infiltrates. The patient was transfused 2 units of packed red blood cells (pRBCs) with improvement of hemoglobin level; however, the hemoglobin level kept declining, and needing repeated pRBCs transfusion. Platelet levels plummeted to the 20s also requiring platelet transfusions. Stool occult blood was repeatedly negative. Blood cultures were negative, but the patient kept spiking fever intermittently (100–105°F). A CT of the abdomen and pelvis revealed multiple enlarged lymph nodes in the chest, abdomen and pelvis, and hepatosplenomegaly. Further testing revealed a ferritin of 7953 ng/mL, IL-2 receptor alpha interleukin 8592, EBV PCR <100 copies/mL, and CMV PCR neg. Bone marrow biopsy of the posterior iliac crest was consistent with hemophagocytosis (Figure 1). An excisional biopsy of an axillary lymph node showed HHV-8 associated multicentric Castleman disease with plasmablastic aggregates and Kaposi sarcoma on CD 138 stain (Figure 2). The patient continues HAART and was started on dexamethasone and etoposide therapy. Unfortunately, patient's condition continued to deteriorate, and family decided to get a palliative consult and place the patient on comfort care until the patient expired.

## 3. Discussion

The diagnosis of HLH is made by fulfilling the following HLH-2004 trial criteria which includes molecular identification of an HLH-associated gene mutation or fulfilling at least 5 out 8 criteria (fever ≥38°C, splenomegaly, peripheral blood cytopenias, hypertriglyceridemia, hemophagocytosis in bone marrow, spleen, lymph node, or liver, low or absent NK cell activity, ferritin >500 ng/mL, elevated soluble CD25 (i.e., soluble IL-2R) at least 2400 U/ml) [2, 4]. The nonspecific nature of the symptoms of HLH delays diagnosis of this life-threatening condition. In this patient, the time period taken to rule out other possible infectious causes of the patient's presentation added to the reluctance in proceeding with more invasive diagnostic procedures like bone marrow biopsy may have led to delayed diagnosis. At the time treatment was started, patient's condition had already deteriorated, making a case for being meticulous because of the toxic nature of medications used. There was also a low suspicion because the patient had been on HAART therapy and was relatively stable. However, a few cases of HLH have been reported in HIV patients who had normal CD 4 counts [1, 2]. The literature also showed that patients who had HLH in HIV had an underlying infection in 40% of cases [7, 8]. This patient had an unremarkable EBV and CMV viral PCR test; however, HHV-8 was found in biopsy. A mortality as high as 31% is reported through an improvement from pre-HAART era with 50–100% mortality [8], and prognosis remains poor [3, 8]. Therapy with etoposide-based chemotherapy has been proposed for treatment [4, 9]. This medication can cause toxicity-related pancytopenia and may worsen outcome in an already advanced patients [4]. This case report suggests that HLH can occur at any stage of HIV and that rapid diagnosis to identify possible underlying reactive infectious etiology and prompt initiation of treatment is crucial for survival.
